# Intravesical Botulinum Toxin for Persistent Autonomic Dysreflexia in a Pediatric Patient

**DOI:** 10.1155/2016/4569684

**Published:** 2016-02-23

**Authors:** Gina Lockwood, Charles Durkee, Travis Groth

**Affiliations:** ^1^Department of Urology, Medical College of Wisconsin, 9000 West Wisconsin Avenue, Milwaukee, WI 53226, USA; ^2^Department of Pediatric Urology, Children's Hospital of Wisconsin, 8915 West Connell Court, Milwaukee, WI 53226, USA

## Abstract

*Introduction*. We present a novel case of persistent autonomic dysreflexia in a pediatric spinal cord injury patient treated successfully with intravesical botulinum toxin.* Study Design*. A retrospective chart review of one patient seen at the Children's Hospital of Wisconsin from 2006 to 2012 was performed.* Results*. A pediatric spinal cord injury patient with known neurogenic bladder presented with severe hypertension consistent with autonomic dysreflexia. His symptoms and hypertension did not improve with conservative measures, and he necessitated ICU admission and antihypertensive drips. He was taken to the operating room for intravesical botulinum toxin for refractory bladder spasms. Following this, his symptoms abated, and he was weaned off IV antihypertensives and returned to his baseline state. His symptoms were improved for greater than six months.* Conclusions*. There are few treatment options for the management of refractory autonomic dysreflexia. Intravesical botulinum toxin has never been reported for this use. Dedicated research is warranted to assess its efficacy, as it was used successfully to abort autonomic dysreflexia in this patient.

## 1. Introduction

Autonomic dysreflexia (AD) can be a life-threatening complication of spinal cord injury (SCI), and management is largely supportive with removal of underlying noxious stimuli. We present a case of a pediatric SCI patient, which illustrates the use of intravesical botulinum toxin (BTX) for treatment of AD refractory to conservative management. A review of the relevant basic science literature is presented, highlighting the mechanism by which BTX may alleviate AD.

## 2. Case

An 11-year-old boy was initially seen in consultation for neurogenic bladder secondary to T4 paraplegia sustained after a motor vehicle collision. Urodynamics were obtained, consistent with neurogenic detrusor overactivity (NDO), detrusor-sphincter dyssynergia (DSD), and symptoms of AD associated with filling. A clean intermittent catheterization (CIC) regimen was initiated for bladder management, with which he was compliant. He reported intermittent symptoms of neck tightness, headache, palpitations, and diaphoresis at home. Blood pressure in the clinic was recorded as high as 190/120 with these symptoms. Constipation, infection, and urinary retention were excluded as causes of AD, and he was managed with oral antihypertensive medications. Over time, he required increasing doses of anticholinergic medications (15 mg oxybutynin extended release formulation daily), for persistent incontinence between catheterizations and worsening bladder spasms. At age twelve, he underwent intravesical BTX injection (300 units). Urodynamics performed three weeks after this revealed neither NDO nor evidence of AD. He underwent five injections over a three-year period with improvement in his symptoms. Less frequent but persistent AD was medically managed with oral labetalol and terazosin, as well as nifedipine and Nitro-Bid cream as needed.

The patient presented to the emergency department at age fifteen with uncontrolled symptoms of AD over five days. His last BTX injection had been just over six months prior to this presentation. Blood pressure was 160/100 on oral antihypertensives. Urinary tract infection and constipation were ruled out as causes, and conservative management with bladder decompression failed to alleviate his symptoms. He was subsequently admitted to the ICU for hemodynamic monitoring. He was placed on an intravenous nifedipine drip. On the day following admission, he went to the operating room for intravesical BTX injection for uncontrolled bladder spasms. This was performed via the standard approach with 300 units injected. On postoperative day two, ICU staff began to wean his antihypertensives. He continued to improve clinically, and headaches and flushing abated. By postoperative day six, the patient was hemodynamically at his baseline, and he was stable for discharge to home on his prehospitalization medications and CIC regimen. Symptoms of AD and bladder spasms were improved for greater than six months.

## 3. Discussion

AD is intimately related to neurogenic bowel and bladder dysfunction, and it can cause life-threatening complications in SCI patients. The mainstay of treatment for neurogenic bladder is CIC, with or without the use of anticholinergics, to curb NDO and maintain low filling and voiding pressures for renal protection [1]. Intravesical BTX is also now routinely used for neurogenic bladder. It has been shown to reduce number of incontinence episodes, as well as detrusor pressure and number of detrusor contractions with filling on urodynamics in both adults and children with NDO [1, 2]. Its use has not been described for management of AD. Traditional treatment for AD specifically aims to quickly remove the precipitating cause, control symptoms, and prevent complications from hypertension [3]. It is rare that AD cannot be corrected by straightforward measures like bladder decompression. Our patient necessitated an intense antihypertensive regimen to control his blood pressure, but the root of his AD was likely bladder spasms from NDO, which could not be controlled conservatively. The effects of BTX on his bladder were not immediate, but while he was under close monitoring and blood pressure control as an inpatient, his overactivity and dysreflexia were eventually eliminated. We propose that intravesical BTX is a potential treatment option for refractory AD associated with NDO. As our understanding expands regarding the physiologic changes that occur after SCI in the spinal cord and bladder, BTX seems a logical candidate for use in AD.

After SCI above the lumbosacral spine, the injured patient initially and immediately experiences complete bladder areflexia (spinal shock). Symptoms of NDO are not seen until 2–12 weeks after injury, causing detrusor overactivity and resultant bladder spasms and urinary incontinence. Accompanying these changes are simultaneous contractions of the bladder and urethral sphincter (DSD) leading to inefficient and incomplete voiding and potentially upper urinary tract deterioration [4, 5]. AD also can develop gradually after high thoracic or cervical SCI, traditionally presenting as paroxysmal hypertension, headache, facial flushing, piloerection, bradycardia or sometimes tachycardia, and profuse sweating above the level of injury [6]. AD may be triggered by activation of pain receptors or from distention of a hollow viscus below the level of SCI. A genitourinary cause is responsible 81–87% of the time [3]. Other less frequent causes than full bladder include full rectum, high bowel impaction, ureteral calculi, fractures of long bones, or perforated abdominal viscera [7]. With distention of the pelvic viscera, massive sympathetic discharge from the splanchnic system between T5 and L3, below the level of the lesion, is initiated reflexively, resulting in vasoconstriction of the muscular, splanchnic, and cutaneous vasculature. Resulting hypertension produces a baroreceptor-mediated reflex bradycardia and vasodilation above the level of the lesion [8]. In a patient without SCI, compensatory vasodilation in the splanchnic bed occurs from higher neural input. In patients with SCI above the level of the sympathetic outflow, the inhibitory pathways are unable to reach the splanchnic bed below the cord lesion. Failure to recognize and treat the problem may result in hypertensive seizures, retinal detachment, or stroke [3]. Treatment algorithms are directed at removal of inciting stimulus (bladder decompression), as well as prevention and termination of hypertension (nifedipine, nitrates, and *α*-adrenergic blockers), as most patients will respond to these measures. Despite potential serious consequences, there are relatively few options for AD refractory to these treatments outside of urinary diversion or sacral denervation [9].

Neuroplasticity is implicated in causing both NDO and AD, mediated by reformation of spinal reflex arcs and bladder afferent neurons following SCI. Animal models have allowed for analysis of their mechanisms, including injury-induced plasticity of primary afferent pathways and changes in electrophysiology of sympathetic and interneurons [10]. Afferent innervation to the bladder arises from the dorsal root ganglia, and nerves supplying the bladder are primarily composed of small, myelinated A-*δ* fibers and unmyelinated C fibers. A-*δ* fibers respond to bladder distention and noxious stimuli to the bladder. C fibers have a high threshold to respond to increased intravesical pressure, but their activity can be enhanced by chemical irritation of the bladder mucosa. Based on animal studies, in a normal spinal cord, the A-*δ* fibers are responsible for the normal feelings of bladder distention as well as the generation of storage and voiding reflexes. However, after SCI, C fiber bladder afferents have been found to initiate voiding [4]. Disruption of descending pathways likely results in local release of neurotrophic factors that induce nerve sprouting, growth, and remodeling of these C fibers. It is these changes that are postulated to play a role in the development of both NDO and AD, causing aberrant physiologic responses to bladder filling and irritation [4, 11].

Nerve growth factor (NGF) is one of the neurotrophic factors implicated, a known mediator of C fiber nerve excitability and bladder reflex arc reconstruction following SCI [12]. Increased NGF has been demonstrated in the bladder, dorsal root ganglia, and spinal cord following SCI, and administration of exogenous NGF to the bladder or spinal cord can in animals induce detrusor overactivity and increase bladder afferent neuron excitability [4, 13]. NGF has been implicated as a urinary biomarker of detrusor overactivity and also has been shown to induce AD in animal models of SCI [14–18]. Krenz et al. proposed a model of AD in which increased NGF in the injured cord stimulates primary afferent nerve sprouting, thus amplifying spinal sympathetic reflexes and promoting dysreflexia [19]. Because of findings like this, contemporary treatments for neurogenic bladder have been aimed at altering conduction through afferent C fibers and the concentration of NGF in the spinal cord. BTX has been the focus of some of these studies.

In August 2011 intravesical BTX was approved by the FDA for urinary incontinence secondary to neurogenic bladder. It has been described as safe and effective both in the children with NDO and in neurologically normal children [20–25]. It is hypothesized that BTX inhibits NDO at both the efferent and afferent limb of the micturition reflex by two entirely different pathways [26]. In addition to the known function of BTX inhibiting vesicular acetylcholine release from the presynaptic nerve terminal at the neuromuscular junction, studies have shown that it is able to inhibit neuropeptide release from primary afferent nociceptive C fibers [27]. BTX A has been shown in multiple studies to reduce NGF concentration in bladders of those with NDO and idiopathic DO [13, 28, 29]. Elkelini et al. showed a direct effect of BTX on AD in animal models, not only decreasing the concentration of NGF but also decreasing number of episodes of AD [30].

Use of BTX for treatment of AD refractory to conservative management has not been described. We have presented here one case of severe AD in a pediatric SCI patient successfully treated with intravesical BTX injection. The common pathways and recent data in the above discussion suggest that BTX is a logical treatment option not only for NDO but for AD as well. Larger, prospective trials are needed to fully assess the efficacy and safety of BTX for this purpose.
